# Relationship between Weight, Body Mass Index, and Bone Mineral Density in Men Referred for Dual-Energy X-Ray Absorptiometry Scan in Isfahan, Iran

**DOI:** 10.1155/2013/205963

**Published:** 2013-10-03

**Authors:** Mohammad Reza Salamat, Amir Hossein Salamat, Iraj Abedi, Mohsen Janghorbani

**Affiliations:** ^1^Department of Medical Physics and Medical Engineering, Isfahan University of Medical Sciences, Isfahan 8144503500, Iran; ^2^Isfahan Osteoporosis Diagnosis and Body Composition Center, Isfahan 8143995518, Iran; ^3^Department of Epidemiology, Isfahan University of Medical Sciences, Isfahan 8144503500, Iran

## Abstract

*Objective.* Although several studies have investigated the association between body mass index (BMI) and bone mineral density (BMD), the results are inconsistent. The aim of this study was to further investigate the relation between BMI, weight and BMD in an Iranian men population. *Methods.* A total of 230 men 50-79 years old were examined. All men underwent a standard BMD scans of hip (total hip, femoral neck, trochanter, and femoral shaft) and lumbar vertebrae (L2-L4) using a Dual-Energy X-ray Absorptiometry (DXA) scan and examination of body size. Participants were categorised in two BMI group: normal weight <25.0 kg/m^2^ and overweight and obese, BMI ≥ 25 kg/m^2^. *Results.* Compared to men with BMI ≥ 25, the age-adjusted odds ratio of osteopenia was 2.2 (95% CI 0.85, 5.93) and for osteoporosis was 4.4 (1.51, 12.87) for men with BMI < 25. It was noted that BMI and weight was associated with a high BMD, compatible with a diagnosis of osteoporosis. *Conclusions.* These data indicate that both BMI and weight are associated with BMD of hip and vertebrae and overweight and obesity decreased the risk for osteoporosis. The results of this study highlight the need for osteoporosis prevention strategies in elderly men as well as postmenopausal women.

## 1. Introduction

Obesity and osteoporosis are two important and growing public health problems worldwide [1–3], and osteoporotic fractures are among the main concerns of elderly population. Low bone mineral density (BMD) is a major risk factor for osteoporosis and its related fractures [3]. Relationship between body mass index (BMI), weight, height, and BMD was reported for many populations [4–6]. Body weight or BMI has been found to be inversely related to the risk of osteoporotic fracture [3, 7]. BMD appears to be reduced in lean postmenopausal women in most [8–18] but not all studies [4, 19–22]; in some studies BMD was reduced [4, 20, 23, 24], whereas in other studies BMD was increased [8–15, 22]. Thus, the role of obesity as a risk factor for low BMD, osteoporosis, and its related fractures remains unsettled. While there have been abundant epidemiological studies in postmenopausal women, few studies have examined the relationship between BMI, weight, and BMD in men and no study has been undertaken in Iranian men. Different associations may be expected in men who have a different lifestyle, such as different levels of activity and different eating habits. However, from clinical and public health point of view, it is important to clarify the role of BMI and weight in association with BMD. Our study contributes to this issue by examining the relationship between BMI, weight, and BMD, among men referred for dual-energy X-ray absorptiometry (DXA) scan in Isfahan, Iran. Our hypothesis is that BMI and weight contribute to the etiology of low BMD. 

## 2. Subjects and Methods

### 2.1. Subjects

This is a cross-sectional study comprised of 230 consecutive not institutionalised men who were referred to Isfahan Osteoporosis Diagnosis and Body Composition Center for DXA scan from May to November 2011, with a mean (standard deviation (SD)) age of 62.6 (8.1) (range 50–79) years. All men were in good health according to clinical medical evaluations. Men who reported chronic medical conditions or were using medications affecting bone metabolism or had family history of osteoporosis and smokers were excluded. Tenets of the current version of Declaration of Helsinki were followed, institutional ethical committee approval was granted, and an informed consent form was signed by each participant.

### 2.2. Anthropometric Measurement

With the subjects in light clothes and without shoes, height and weight were measured using standard apparatus while performing bone densitometry measurements. Weight was measured to the nearest 0.1 kg on a calibrated beam scale. Height was measured to the nearest 0.5 cm with a measuring tape. We calculated BMI as the ratio of weight (kg) to height squared (m^2^). Participants were categorised in two BMI groups according to World Health Organisation (WHO) criteria [25]: normal weight, BMI < 25.0 kg/m^2^; overweight and obese, BMI ≥ 25 kg/m^2^.

### 2.3. Bone Density Measurement

Measurements of BMD (g/cm^2^) and bone mineral content (BMC) (g) were made using DXA (Norland XR-46 system; Coopersurgical, Fort Atkinson, WI, USA). BMDs of the lumbar vertebrae (L2–L4) and the hip region (total hip, femoral neck, trochanter, and femoral shaft) were measured according to protocols. The scanner was calibrated daily against the standard calibration block supplied by the manufacturer to control for possible baseline drift. T-Score and Z-Score were also obtained. The diagnosis of osteoporosis/osteopenia was done according to T-score values: normal if T-score ≥ −1.0; osteopenia if −2.5 < T-score < −1.0; osteoporosis if T-score ≤ −2.5 [26]. All the data were collected according to the recommendations of the International Society for Clinical Densitometry [27].

### 2.4. Analysis

Statistical methods used included the Pearson's correlation; chi-squared test, one-way analysis of variance, multiple linear regression, and binary logistic regression. Pearson's correlation was used to measure the correlation between continuous variables. Multivariate binary logistic regressions were fitted to identify predictors of osteoporosis/osteopenia. Variable age was entered in models as continuous variable while BMI was categorical. When multiple linear regression analysis was used, BMDs and BMCs in the lumbar vertebrae, total hip, femoral neck, femoral shaft, and trochanter were dependent variables, whereas age, BMI, and weight were independent variables. Age-adjusted means were also calculated and compared using general linear models. All anthropometric or DXA measures were not included simultaneously in regression analysis to avoid colinearity that these independent variables may have. All tests for statistical significance were two-tailed and performed assuming a type I error probability of <0.05. SPSS version 18.0 for Windows (SPSS Inc., Chicago, IL, USA) was used for the statistical analysis.

## 3. Results

### 3.1. Subject Characteristics

Characteristics of the 95 (41.3%) participants with BMI < 25 kg/m^2^ and 135 (58.7%) with BMI ≥ 25 are shown in Table 1. Those who had BMI < 25 were older; had lower age-adjusted mean BMI, weight, lumbar vertebrae, total hip, femoral neck, and trochanteric BMDs and BMCs, and have higher proportion of osteoporosis. No significant difference was observed for height. The mean (SD) age was 63.9 (7.9) years for those with BMI < 25 and 61.7 (8.1) years for those with BMI ≥ 25. The mean (SD) BMI was 22.6 (1.7) kg/m^2^ for those with BMI < 25 and 28.5 (2.4) kg/m^2^ for those with BMI ≥ 25. A total of 96 (41.7%) of the men were overweight (BMI > 25 and < 30) and 36 (15.6%) were obese (BMI ≥ 30 kg/m^2^).

The means and standard deviations of anthropometric and densitometric measures by age decade and BMI class are shown in Table 2. There was a decrease in weight and height with age in both men with BMI < 25 and BMI ≥ 25 kg/m^2^. A significant decrease in BMI with age was observed only when all BMI groups were considered (*P* < 0.05). A significant decrease in femoral neck with age was observed in both groups. A significant decrease in total hip and femoral shaft BMD with age was observed only in overweight and obese men and when all BMI groups were considered (*P* < 0.01).

### 3.2. Prevalence of Low Bone Density

The results of the DXA scan show only 28 (12.2%) men had normal DXA scan results. Of the 95 men who had BMI < 25 kg/m^2^, 6 (6.3%, 95% CI: 1.4, 11.2) had normal BMD. This was lower than the percentage seen for men with BMI ≥ 25 kg/m^2^, 16.3% (95% CI: 10.1, 22.5). When measurements at lumbar vertebrae and hip regions were considered simultaneously, a total of 144 cases of osteopenia and 58 cases of osteoporosis were diagnosed. The overall prevalence of osteopenia was 62.6% (95% CI: 56.4, 68.9). Of the 95 men who had BMI < 25 kg/m^2^, 56 had osteopenia, giving a prevalence of 58.9% (95% CI: 48.4, 68.9). Although this was lower than the prevalence rates seen for those who had BMI ≥ 25, 65.2% (95% CI: 57.1, 73.2), the difference was not statistically significant (*P* > 0.05). The overall prevalence of osteoporosis was 25.2% (95% CI: 19.6, 30.8). Of the 95 men who had BMI < 25 kg/m^2^, 33 had osteoporosis, giving a prevalence of 34.7% (95% CI: 25.3, 45.2). This was higher than the prevalence rates seen for men with BMI ≥ 25 kg/m^2^, 18.5% (95% CI: 12.0, 25.1) (*P* < 0.05) (Table 3).

### 3.3. Risk Factors

Compared with men with BMI≥ 25 kg/m^2^, the age-adjusted risk of osteoporosis was over four-fold higher in those with BMI < 25 (odds ratio (OR) 4.4 (1.51, 12.87) in age-adjusted model (Table 3). The association between BMI and osteopenia was similar among overweight/obese and normal weight men, although not statistically significant (Table 3).

Both BMI and weight were correlated with BMD, and BMC indicators and the strongest Pearson's correlation coefficients were found between weight and BMC in total hip and trochanter regions and the weakest ones were between BMI and femoral neck BMC. The correlations between age and BMD and BMC indicators were negative (Table 4).

The age-adjusted proportion of BMD and BMC variation explained by BMI and weight is shown in Table 5. Weight tended to show greater differences in BMD and BMC distribution at all the studied skeletal sites as compared to BMI. This was reflected by the steeper slope (*β*) of the respective linear regression lines. The greatest difference in BMD and BMC distribution appeared at the trochanter BMC followed by lumbar BMC for weight. Weight is an important predictor of BMD and BMC variation in elderly Iranian men.

## 4. Discussion

In this study, both BMI and weight were associated with BMD, and obesity significantly decreased the risk for osteoporosis in men. The relationship between BMD and weight was stronger than between BMI and BMD. These results are consistent with most previous studies, particularly in postmenopausal women, which indicated that lower BMI and weight were associated with lower BMD. Prospective studies found that the early postmenopausal women who have low BMI lose more bones compared to those with higher BMI tertiles [4, 8]. In other cross-sectional studies, however, thinness is related to both osteoporosis and increased fracture risk [10, 11]. Iqbal et al. [9] found that low BMI is a good indicator for referral of women less than 60 years old for measurements of BMD. Similar studies also reported a consistent finding that lower BMI was associated with lower BMD [5, 6, 28]. In a study similar to ours, those with lower BMI were at higher risk of low BMD [18]. The Studies of National Osteoporosis Foundation and others suggested that low BMI should be included in the risk assessment tools for evaluation of osteoporosis and osteoporotic fracture risk [29–31]. We found that men with BMI < 25 had 4.4 (95% CI 1.5, 12.8) times higher age-adjusted risk of osteoporosis than men with BMI ≥ 25. In contrast, Steinschneider et al. [32] in a cross-sectional study reported that the correlation between BMD at the femoral neck and BMI was highly positive among postmenopausal women. A hospital-based study conducted in elderly men reported that overweight and obese men were more likely to have osteoporosis and osteopenia [33]. The possible explanation for the discrepancies between these results might be related to populations, research designs, sampling methods, and methodological differences.

The mechanisms whereby adipose tissue exerts positive effects on BMD status are not entirely clear. The putative mechanism relevance of adipose tissue for skeletal integrity probably resides in the role of several adipokines in bone remodeling through effect on both formation and resorption. Recently, bone has been considered an endocrine organ affecting body weight control and glucose homoeostasis through the actions of bone-derived factors such as osteocalcin and osteopontin [23, 34–36]. The putative crosstalk between fat and the skeleton constitutes a homoeostatic feedback system in which adipokines and molecules secreted by osteoblasts and osteoclasts represent the link of an active bone-adipose axis [34–36]. Obesity is also associated with BMD because of the conversion of androgen to estrogen [37], which improves bone mass in both men and women [38, 39] and maintains healthy plasma levels of insulin and regulating factors including insulin-like growth factor-1, leptin, and adiponectin [40]. In addition, obesity provides cushioning for the hip in the event of a fall [3]. However, the mechanisms by which all these events occur remain unclear.

Although this study had several findings relevant to the better understanding of the relationship between weight, BMI, and BMD in an Iranian men population, it has some limitations. One potential source of bias in the present study is residual confounding due to the risk factors that we were unable to account for in our analysis (socioeconomic status, educational level, level of physical activity, smoking, alcohol consumption, vitamin D status, sex hormone levels, and nutrition). Moreover, unknown confounders cannot be adjusted for. Thus, the observed decreased risk of osteoporosis associated with BMI may reflect confounding by these risk factors. The study was clinic-, rather than population-, based and so may not contain a clinical spectrum representative of elderly men in the community. Clinic-based estimates of the prevalence of low bone mass are most likely to be affected by referral patterns. Selection bias is less likely to affect associations between BMI, and BMD as investigated in this study. As a cross-sectional study, the present analysis is limited in its ability to elucidate causal relationships between weight, BMI and BMD. Another limitation was that study participants were 50–80 years, and results may not apply to the broader age groups. Nevertheless, this study provides new data from Iran, a developing country, which has been underrepresented in past studies.

In summary, our study indicates that both BMI and weight are associated with BMD of hip and vertebrae regions and overweight and obesity decreased the risk for osteoporosis. These findings highlight the need for osteoporosis prevention efforts in elderly men as well as postmenopausal women.

## Figures and Tables

**Table 1:** Age, age-adjusted means (SD), and proportions of selected anthropometric and densitometric measures between 95 men with body mass index (BMI) < 25 kg/m^2^ and 135 men who had BMI ≥ 25.

Variables	BMI < 25 kg/m^2^	BMI ≥ 25 kg/m^2^	Difference (95% CI)
	Mean (SD)	Mean (SD)	
Age (yr.)	63.9 (7.9)	61.7 (8.1)	2.2 (0.08, 4.3)*
Height (cm)	168.1 (6.4)	168.3 (5.8)	−0.2 (−1.40, 1.80)
Weight (kg)	63.9 (7.1)	80.6 (8.6)	−16.7 (−18.50, −14.30)***
BMI (Kg/m^2^)	22.6 (1.7)	28.4 (2.4)	−5.8 (−6.46, −5.34)***
L2–L4 BMD (g/cm^2^)	0.99 (016)	1.09 (0.17)	−0.1 (−0.14, −0.06)***
Femoral neck BMD (g/cm^2^)	0.79 (0.12)	0.82 (0.12)	−0.03 (−0.07, −0.002)*
Trochanter BMD (g/cm^2^)	0.69 (0.10)	0.73 (0.11)	−0.04 (−0.06, −0.01)**
Femoral shaft BMD (g/cm^2^)	1.01 (0.13)	1.07 (0.15)	−0.06 (−0.10, −0.02)**
Total hip BMD (g/cm^2^)	0.88 (0.11)	0.94 (0.13	−0.06 (−0.09, −0.03)**
L2–L4 BMC (g)	50.0 (9.7)	56.0 (9.6)	−6.0 (−8.54, −3.46)***
Femoral neck BMC (g)	4.3 (0.8)	4.5 (0.7)	−0.20 (−0.40, −0.002)*
Trochanteric BMC (g)	10.6 (1.9)	11.6 (2.3)	−1.00 (−1.57, −0.43)***
Femoral shaft BMC (g)	17.3 (2.4)	18.8 (2.9)	−1.50 (−2.01, −0.59)***
Total hip BMC (g)	33.6 (4.5)	36.2 (5.3)	−2.6 (−3.92, −1.28)***
L2–L4 T-score	−1.3 (0.8)	−0.8 (0.8)	−0.50 (−0.71, −0.29)***
Femur neck T-score	−2.6 (0.9)	−2.3 (1.0)	−0.30 (−0.55, −0.05)*
Trochanter T-score	−2.2 (0.8)	−1.8 (1.0)	−0.40 (−0.54, −0.06)**
Total hip T-score	−2.1 (0.9)	−1.7 (1.1)	−0.4 (−0.67, −0.13)**

	No. (%)	No. (%)	

Osteoporosis	33 (34.7)	25 (18.5)	16.2 (4.62, 27.80)**
Osteopenia	56 (58.9)	88 (65.2)	−6.3 (−19.00, 6.51)
Normal weight (BMI < 25)	95 (100.0)	—	−
Overweight (BMI ≥ 25 < 30)	—	96 (71.1)	−71.1 (−78.8, −63.5)***
Obese (BMI ≥ 30)	—	39 (28.9)	−28.9 (−36.5, −21.2)***

Age-adjusted means were calculated using general linear models. The difference in the mean or percentage of the variables between BMI < 25 and BMI ≥ 25. *P < 0.05, **P < 0.01, and ***P < 0.001. CI: confidence interval.

**Table 2:** Anthropometric and densitometric measures of men aged 50 years and older by age group and BMI classification.

	Age (year) Mean (SD)			
	50–59	60–69	70–79	Total
Anthropometric measures				
BMI < 25.0, no. (%)	34 (35.8)	30 (31.6)	31 (32.6)	95 (100.0)
Weight (kg), mean (SD)	63.5 (6.2)	67.9 (6.0)	60.5 (7.1)***	63.9 (7.1)
Height (cm), mean (SD)	168.2 (5.6)	171.7 (5.8)	164.5 (5.5)***	168.1 (6.4)
BMI (kg/m^2^), mean (SD)	22.5 (1.6)	23.0 (1.6)	22.3 (1.9)	22.6 (1.7)
BMI ≥ 25 no. (%)	64 (47.4)	46 (34.1)	25 (18.5)	135 (100.0)
Weight (kg), mean (SD)	81.6 (7.8)	80.9 (9.6)	77.4 (8.1)**	80.6 (8.6)
Height (cm), mean (SD)	169.9 (5.0)	167.6 (6.4)	165.4 (5.3)**	168.3 (5.8)
BMI (kg/m^2^), mean (SD)	28.3 (2.2)	28.7 (2.6)	28.3 (2.6)	28.4 (2.4)
All BMI group no. (%)	98 (42.6)	76 (33.0)	56 924.3)	230 (100.0)
Weight (kg), mean (SD)	75.4 (11.3)	75.8 (10.5)	68.0 (11.3)***	73.7 (11.5)
Height (cm), mean (SD)	169.3 (5.3)	169.2 (6.5)	164.9 (5.4)***	168.2 (6.0)
BMI (kg/m^2^), mean (SD)	26.3 (3.4)	26.5 (3.6)	25.0 (3.7)*	26.0 (3.6)
Densitometry measures				
BMI < 25.0				
Femoral neck BMD (g/cm^2^)	0.82 (0.12)	0.80 (0.09)	0.73 (0.12)**	0.79 (0.12)
Trochanteric BMD (g/cm^2^)	0.70 (0.10)	0.71 (0.08)	0.66 (0.11)	0.69 (0.10)
Femoral shaft BMD (g/cm^2^)	1.03 (0.12)	1.01 (0.14)	0.98 (0.13)	1.01 (0.13)
L2–L4 BMD (g/cm^2^)	0.97 (0.14)	1.05 (0.15)	0.98 (0.18)	1.00 (0.16)
Total hip BMD (g/cm^2^)	0.90 (0.10)	0.90 (0.10)	0.85 (0.11)	0.88 (0.11)
BMI ≥ 25				
Femoral neck BMD (g/cm^2^)	0.85 (0.12)	0.83 (0.11)	0.73 (0.11)***	0.82 (0.12)
Trochanteric BMD (g/cm^2^)	0.74 (0.11)	0.75 (0.10)	0.69 (0.15)	0.73 (0.11)
Femoral shaft BMD (g/cm^2^)	1.09 (0.16)	1.10 (0.13)	0.99 (0.17)**	1.07 (0.15)
L2–L4 BMD (g/cm^2^)	1.07 (0.16)	1.09 (0.16)	1.16 (0.19)	1.09 (0.17)
Total hip BMD (g/cm^2^)	0.95 (0.13)	0.96 (0.10)	0.87 (0.15)*	0.94 (0.13)
All BMI group				
Femoral neck BMD (g/cm^2^)	0.84 (0.12)	0.82 (0.10)	0.73 (0.12)***	0.80 (0.12)
Trochanteric BMD (g/cm^2^)	0.73 (0.11)	0.73 (0.09)	0.67 (0.13)**	0.72 (0.11)
Femoral shaft BMD (g/cm^2^)	1.07 (0.15)	1.07 (0.14)	0.98 (0.15)**	1.05 (0.15)
L2–L4 BMD (g/cm^2^)	1.03 (0.16)	1.07 (0.16)	1.06 (0.20)	1.05 (0.17)
Total hip BMD (g/cm^2^)	0.93 (0.12)	0.93 (0.11)	0.86 (0.13)***	0.91 (0.12)

*P < 0.05,  **P < 0.01, and  ***P < 0.001.

**Table 3:** Prevalence rates and odds ratio (95% CI) of osteopenia and osteoporosis by body mass index status.

Variables	Cases (no.)	Prevalence (%) (95% CI)	Age-adjusted odds ratio (95% CI)^†^
Osteopenia	144	62.6 (56.4, 68.9)	—
BMI ≥ 25	88	65.2 (57.1, 73.2)	1.00
BMI < 25	56	58.9 (48.4, 68.9)	2.2 (0.85, 5.93)
Osteoporosis	58	25.2 (19.6, 30.8)	
BMI ≥ 25	25	18.5 (12.0, 25.1)	1.00
BMI < 25	33	34.7 (25.3, 45.2)	4.4 (1.51, 12.87)*

^†^Odds ratio (with 95% CI) calculated by multiple logistic regression.  *P < 0.05. CI: confidence interval.

**Table 4:** Pearson's correlation coefficients between BMI, weight, age, and BMD and BMC indicators.

Variables	Body mass index (kg/m^2^)	Weight (kg)	Age (year)
L2–L4 BMD (g/cm^2^)	0.331***	0.324***	0.05
Femoral neck BMD (g/cm^2^)	0.189***	0.280***	−0.361*
Trochanteric BMD (g/cm^2^)	0.296***	0.357***	−0.223**
Femoral shaft BMD (g/cm^2^)	0.296***	0.333***	−0.048
Total hip BMD (g/cm^2^)	0.286***	0.328***	−0.253***
L2–L4 BMC (g)	0.344***	0.454***	0.002
Femoral neck BMC (g)	0.182***	0.305***	−0.263*
Trochanteric BMC (g)	0.334***	0.505***	−0.166*
Femoral shaft BMC (g)	0.319***	0.351***	−0.130*
Total hip BMC (g)	0.348***	0.458***	−0.179**

*P < 0.05, **P < 0.01, and ***P < 0.001.

**Table 5:** Age-adjusted linear regression analysis for BMD and BMC with BMI and weight as regressor.

Variables	Body mass index (kg/m^2^)		Weight (kg)	
	*β*	*R* ^2^ (%)	*β*	*R* ^2^ (%)
L2–L4 BMD (g/cm^2^)	0.335***	0.11	0.345***	0.12
Femoral neck BMD (g/cm^2^)	0.165**	0.16	0.220***	0.18
Trochanteric BMD (g/cm^2^)	0.281***	0.13	0.327***	0.15
Femoral shaft BMD (g/cm^2^)	0.280***	0.13	0.299***	0.14
Total hip BMD (g/cm^2^)	0.270***	0.14	0.290***	0.14
L2–L4 BMC (g)	0.346***	0.12	0.471***	0.21
Femoral neck BMC (g)	0.164*	0.10	0.264***	0.14
Trochanteric BMC (g)	0.324***	0.12	0.491***	0.26
Femoral shaft BMC (g)	0.312***	0.11	0.338***	0.13
Total hip BMC (g)	0.337***	0.14	0.440***	0.21

*P < 0.05, **P < 0.01, and ***P < 0.001.
